# The effect of foot orthoses with forefoot cushioning or metatarsal pad on forefoot peak plantar pressure in running

**DOI:** 10.1186/s13047-016-0176-z

**Published:** 2016-11-16

**Authors:** Michaela Hähni, Anja Hirschmüller, Heiner Baur

**Affiliations:** 1 Bern University of Applied Sciences, Health, Physiotherapy, Murtenstrasse 10, 3008 Bern, Switzerland; 2 University Hospital Freiburg, Clinic for Orthopaedics and Traumatology, Hugstetter Strasse 55, 79106 Freiburg, Germany

**Keywords:** Foot orthoses, Peak plantar pressure, Forefoot, Running

## Abstract

**Background:**

Foot orthoses are frequently used in sports for the treatment of overuse complaints with sufficient evidence available for certain foot-related overuse pathologies like plantar fasciitis, rheumatoid arthritis and foot pain (e.g., metatarsalgia). One important aim is to reduce plantar pressure under prominent areas like metatarsal heads. For the forefoot region, mainly two common strategies exist: metatarsal pad (MP) and forefoot cushioning (FC). The aim of this study was to evaluate which of these orthosis concepts is superior in reducing plantar pressure in the forefoot during running.

**Methods:**

Twenty-three (13 female, 10 male) asymptomatic runners participated in this cross-sectional experimental trial. Participants ran in a randomised order under the two experimental (MP, FC) conditions and a control (C) condition on a treadmill (2.78 ms^−1^) for 2 min, respectively. Plantar pressure was measured with the in-shoe plantar pressure measurement device pedar-x®-System and mean peak pressure averaged from ten steps in the forefoot (primary outcome) and total foot was analysed. Insole comfort was measured with the Insole Comfort Index (ICI, sum score 0–100) after each running trial. The primary outcome was tested using the Friedman test (α = 0.05). Secondary outcomes were analysed descriptively (mean ± SD, lower & upper 95%-CI, median and interquartile-range (IQR)).

**Results:**

Peak pressure [kPa] in the forefoot was significantly lower wearing FC (281 ± 80, 95%-CI: 246–315) compared to both C (313 ± 69, 95%-CI: 283–343; *p* = .003) and MP (315 ± 80, 95%-CI: 280–350; *p* = .001). No significant difference was found between C and MP (*p* = .858). Peak pressures under the total foot were: C: 364 ± 82, 95%-CI: 328–399; MP: 357 ± 80, 95%-CI: 326–387; FC: 333 ± 81 95%-CI: 298–368. Median ICI sum scores were: C 50, MP 49, FC 64.

**Conclusions:**

In contrast to the metatarsal pad orthosis, the forefoot cushioning orthosis achieved a significant reduction of peak pressure in the forefoot of recreational runners. Consequently, the use of a prefabricated forefoot cushioning orthosis should be favoured over a prefabricated orthosis with an incorporated metatarsal pad in recreational runners with normal height arches.

**Electronic supplementary material:**

The online version of this article (doi:10.1186/s13047-016-0176-z) contains supplementary material, which is available to authorized users.

## Background

Due to its positive effects on cardiorespiratory and muscular fitness and its accessibility, running is a common recreational sport with increasing popularity [1–3]. According to a survey conducted in 2014, 23.3% of the Swiss population (aged 15 to 74 years) runs at least once a week [4]. In the EU, the total number of runners can be estimated to be 50 million [5] and in the USA more than 40 million people run regularly [6]. Despite the health benefits of running, there is, however, a yearly incidence of 19.4% to 79.3% in runners suffering from a running-related injury [3, 7]. Overuse is an important cause of running injuries, whereas debates continue about the aetiological factors. Possible risk factors reported by several authors are long weekly training distances, history of previous injuries, altered gait biomechanics and foot posture [3, 7–13]. A retrospective study which examined 2002 patients with running-related injuries showed the ankle/foot area to be the second most common overuse injury location after the knee [13]. Such overuse injuries include forefoot overuse complaints like metatarsalgia, stress fractures and plantar fasciopathy [14, 15]. Previous studies of long-distance runners found a significant increase in plantar pressure in the forefoot after compared to before the run [2, 14]. This might explain the risk of running injuries like stress fractures of metatarsals or metatarsalgia in long-distance runners [2, 14].

Foot orthoses have been identified as one potential tool for decreasing the incidence of lower extremity injuries by reducing the magnitude and rate of loading [16, 17]. It has been conclusively shown that foot orthoses can relieve symptoms in certain types of foot-related pathologies, like plantar fasciitis, rheumatoid arthritis and foot pain (e.g., metatarsalgia); moreover, they improve comfort [18–23].

Contoured prefabricated foot orthoses can be used to reduce plantar pressure under the forefoot [24, 25]. In current practice in central Europe, there are generally two main concepts concerning foot orthoses to reduce plantar pressure in the forefoot. One is placing a metatarsal pad proximal to the metatarsal heads and the other one is integrating forefoot cushioning. A previous study investigated the effects of three foot pads (metatarsal pad, U-shaped pad and doughnut-shaped pad) on the plantar pressure of participants with pes planus foot type during running, metatarsal pads being reported to be most effective in significantly reducing peak pressure [26]. This work is complemented by the results of several experimental trials showing that a metatarsal pad (metatarsal pad (centrally just proximal to the 2nd, 3rd and 4th metatarsal heads (MHs)), metatarsal bar (proximal to the MHs), metatarsal dome (5 mm proximal to the 2nd and 4th MHs, 5 mm distal to the MHs)) can significantly reduce plantar pressure on the central and medial forefoot in walking individuals [20, 27–33]. As for the cushioning orthoses, two other studies reported effective reduction of peak pressure in the forefoot during walking and running in military boots [34, 35]. In addition to these two concepts, pressure redistribution to the midfoot by full contact orthoses is used in the Anglo-American area.

Despite the widespread use of orthoses to reduce plantar pressure in the forefoot of runners, no previous studies have investigated the comparison of a forefoot cushioning orthosis and an orthosis with a metatarsal pad. This indicates a need to understand which foot orthosis strategy is best to reduce plantar pressure in the forefoot of runners.

The present study evaluated and compared the effect of forefoot cushioning and a metatarsal pad on peak pressure in the forefoot of asymptomatic recreational runners.

It was hypothesised that there would be no difference in peak pressure in the forefoot between the forefoot cushioning orthosis and the metatarsal pad condition.

## Methods

### Design/Setting

This cross-sectional experimental trial was conducted from December 2013 to May 2015. Every participant had to run on a treadmill (2.78 ms^−1^) in a regular neutral running shoe (model: Duramo 6, Adidas®, Herzogenaurach D, year: 2014) with three different types of foot orthoses (neutral, forefoot cushioning and metatarsal pad) in a randomised order.

### Participants

The descriptive characteristics of the twenty-five recreational runners (15 females and 10 males) participating in this study are shown in Table 1. They had been recruited via e-mail and from the local running community. Criteria for inclusion were as follows: recreational runners (at least two running sessions per week) aged between 18 and 65 years, and being accustomed to treadmill running. The exclusion criteria were forefoot strike running pattern, history of an injury of the lower extremity within the last 6 months before the study, acute disorders in the lower extremities and spine, history of surgery on the lower extremities and lumbar spine in the last 24 months, acute infection and other complaints that would have impeded completing the protocol. Foot characteristics were documented using the normalised navicular height truncated (NNHt) [36], which classified all of the participants’ feet as normal-arched with no significant side difference (see Table 1).

**Table 1:** Participant characteristics

Participant characteristics (*n* = 25)	Mean ± SD
Age (years)	31.7 ± 9.4
Weight (kg)	64.0 ± 8.6
Height (cm)	172.7 ± 8.9
BMI (kg/cm^2^)	21.4 ± 1.8
Training volume (min/week)	327 ± 177
Running volume (min/week)	166 ± 101
NNHt right foot	0.26 ± 0.03

*BMI* body mass index, *NNHt* normalised navicular height truncated, *SD* standard deviation

The study protocol was approved by the Ethics Committee of the Canton of Berne, Switzerland (16.10.2012 Nr. Z039/12) and written consent was obtained from all participants. The study was carried out in accordance with the stipulations of the World Medical Association Declaration of Helsinki [37].

### Orthoses construction

**Fig. 1:**
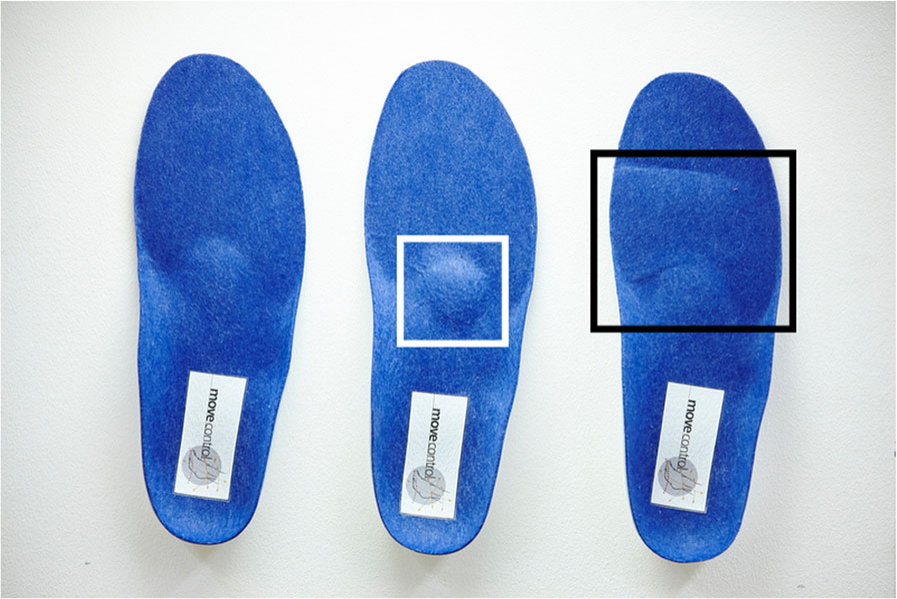
Upper surface of the orthoses used in this study. Left to right: control, metatarsal pad, forefoot cushioning (shore 12)

**Fig. 2:**
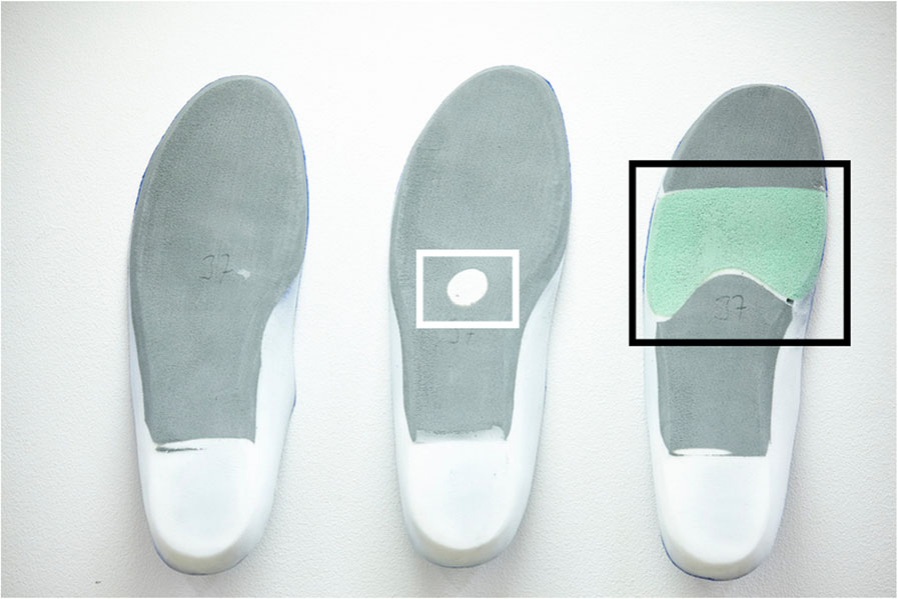
Bottom surface of the orthoses used in this study. Left to right: control, metatarsal pad, forefoot cushioning (shore 12)

The foot orthoses used in this study (Movecontrol®, IETEC®, Künzell, D) were made of polyurethane foam material (shore 25; with an ethylene vinyl acetate EVA core, shore 55, compression moulded and semirigid) and prefabricated. The basic shape (control condition) comprised a concave-shaped heel, a minimal medial longitudinal arch support and a low metatarsal pad (see Fig. 1). The forefoot cushioning pad (PU, shore 12) had a thickness of 6 mm and covered the complete forefoot (Fig. 2 - right). The metatarsal pad (PU) had a size of 5.0 by 5.0 cm with a width towards the forefoot of 5.0 cm and a width towards the mid- and rear foot of 2.5 cm. The height increased from its boarders to a maximum of 8 mm. The basic form of the foot orthoses already had a small pad integrated (3.5 by 3.5 cm, height: 2 mm). This pad is part of all basic foot orthosis forms of this type (Movecontrol®, IETEC®, Künzell, D) and smoothly transfers to the longitudinal arch support. It was implemented in the control condition and in the forefoot cushioning condition. Therefore, the pad condition resulted in a pad with an additional height of 6 mm compared to the control condition (and compared to the forefoot cushioning condition).

The orthoses were selected according to the foot length of each participant. Additionally, an identical blue felt layer of 1 mm was fixed onto each upper surface of the three orthoses (see Fig. 1). This standardised form of fabrication enabled a visual blinding of the participants in terms of the type of orthosis they were running with and provided standardised testing conditions for the measurements. For the application of this type of orthosis in patients, however, the orthoses are made and adapted by customising on an individual basis.

### Procedure

After obtaining written informed consent from the participants, background demographic data (age, weight, height, shoe size, and running and training frequency) were collected and foot measurements for the NNHt were taken. Subsequently, each participant did a warm-up on the treadmill (Kettler Marathon TX 1, Kettler, Ense-Parsit, D) for 6 min at a running speed of 2.78 ms^−1^, wearing a neutral running shoe (model: Duramo 6, Adidas®, Herzogenaurach, D, year 2014), which was fitted beforehand. Afterwards the plantar pressure measurement device (pedar-x®-System (Novel®, München, D)) was mounted on the participants’ feet. The insoles with 99 capacitive sensors resulting in approximately one sensor per 2 cm^2^ (depending on insole size) were placed in the neutral running shoes. The order of testing conditions was randomised using the online tool Randomization.com. None of the participants used this type of foot orthoses during their regular running sessions.

The measurements took 2 min per condition during treadmill running. The first minute served for familiarisation with the orthosis and the second for data collection.

Plantar pressure data were collected for one minute sampled at 100 Hz and the mean peak pressure of ten steps was analysed. Percentage masks were used for definition of forefoot area. The forefoot mask was defined from 62% to 80% (from total foot length, heel: 0%). This ensured exclusion of the metatarsal pad from the forefoot area. To control for possible confounding information about foot orthoses design, sampling of data was performed without informing the participants about the current orthosis condition and the exact moment of testing [38]. After completion of each bout of running, participants had to fill out the Insole Comfort Index (ICI, see below), asking for an individual comfort rating of foot orthosis condition [39, 40].

### Data processing

Data were checked for plausibility with the manufacturer’s measurement software package (pedar®-x Recorder, version 19.3.30, novel®, Munich, D). All files were verified for rearfoot strike running pattern and continuous COP paths in each step. Incomplete steps were removed from further analysis. Finally, data from 10 steps of the right foot (to avoid using paired data) [41] were averaged (Novel® scientific software, version 19.3.42, Novel®, Munich D) to achieve a representative pressure distribution per condition [25]. After checking data for plausibility, data from two participants were excluded (dropouts) because of not having used a rear foot strike running pattern. Thus, 23 runners remained for the final analysis.

The outcome measurements were the following:

1. Primary outcome: Peak plantar pressure in the forefoot area: Measured with the in-sole plantar pressure measurement system pedar-x®-System (Novel®, München, D) a valuable and reliable tool in the assessment of plantar pressure distribution [42–45]. The reliability of this outcome was previously checked in our laboratory using a test-retest design with 17 healthy participants measured one week apart with the same protocol. Forefoot peak pressures showed excellent reliability with an ICC of 0.97, a test-retest variability expressed as a percentage of 4.9 ± 6.5% and a systematic error (bias) of 4.1 kPa with limits of agreement according to Bland and Altman (standard deviation*1.96) of 49.5 kPa [46]. The software pedar®-x-expert (Version 19.3.30, Novel®, München, D) and novel scientific (Version 19.3.42, Novel®, München, D) were applied for data logging and evaluation.
2. Secondary outcomes:
  - Peak plantar pressure in the total foot area: Peak pressure of the total area was extracted from pressure data. Similarly, for total foot peak pressures, reliability was checked as mentioned previously and revealed an ICC of 0.97, a test-retest variability of 4.7 ± 4.5% and a bias of 1.2 kPa with limits of agreement of 49.1 kPa [46]. Data on the total foot were extracted to allow comparison to other studies irrespective of methodological differences to appraise general peak pressure ranges measured.
  - Insole Comfort Index (ICI): Orthosis comfort was rated on five independent (overall comfort, forefoot comfort, heel comfort, roll off comfort and comfort compared to no orthosis) visual analogue scales ranging from “very comfortable” to “not comfortable” [39, 40]. For statistical evaluation, sum scores of all items were calculated [0–100]. ICI based on visual analogue scales (10 cm) is considered to be a reliable tool to assess footwear comfort during running [39].

### Statistical analysis

An a priori analysis of effect size and sample size was made for a desired power of 80% and an α-error of 0.05. Effect size was taken from unpublished pilot data in walking (1.11 ms^−1^) on this topic with the same dependent (forefoot peak pressure) and independent variables (orthosis type). Sample size was calculated using the G*Power 3 software [47] and estimated a minimum sample size of 16 participants.

Prior to the statistical analysis, data from case report forms were entered manually into a prepared database. Data were then checked for normal distribution (Shapiro-Wilk test (*p* > 0.25)). The Shapiro-Wilk test revealed that peak pressure at the forefoot significantly deviates from a normal distribution for the two tested conditions (metatarsal pad: *p* = 0.017, forefoot cushioning: *p* = 0.004). Only pressure data for the normal condition were normally distributed (*p* = 0.061). Nevertheless, mean (SD) and 95% CI of peak pressure data are reported in descriptive statistics in addition to the median (IQR) to compare the results of this study with the current literature. Subsequently, peak pressures for the right forefoot and the total area of the right foot were analysed descriptively with calculation of mean, standard deviation (SD), 95% confidence intervals (95% CI), median and interquartile range (IQR). Finally, the initial hypothesis (no differences in peak pressure in the forefoot between conditions) was tested using the Friedman test (*p* = 0.05) and post hoc Wilcoxon signed-rank test. Post hoc analysis was conducted applying a Bonferroni correction, resulting in a significance level set at *p* < .017. Furthermore, peak pressure of the total foot area and sum scores of ICI were analysed using descriptive statistics (median (IQR)). The statistical analysis was performed using SPSS software version 22.0 (IBM, SPSS, Inc., Chicago, IL, USA).

## Results

### Peak pressure in the forefoot area

Analysis of peak pressure in the forefoot showed a significant difference between the three different conditions of orthosis (*χ* ^2^(2) = 19.143, *p* < .001). The post hoc analysis revealed statistically significant lower peak pressure in the forefoot for the cushioning orthosis compared to both control condition (*p* = .003) and metatarsal pad orthosis (*p* = .001). On average, peak pressure in the forefoot of runners wearing the forefoot cushioning orthosis compared to the control condition was reduced by 11% and compared to metatarsal pad orthosis by 12%, whereas no significant difference in peak pressure in the forefoot was found between the control condition and the metatarsal pad orthosis (*p* = .858).

The results obtained from the descriptive analysis of peak pressure in the forefoot for the three conditions are presented in Table 2.

**Table 2:** Peak pressure [kPa] in the forefoot wearing the three different types of foot orthoses (conditions)

Condition	Mean	SD	Lower 95%-CI	Upper 95%-CI	Median	IQR
control	313	69	283	343	300	258 to 348
pad	315	80	280	350	300	255 to 348
cushioning	281	80	246	315	268	235 to 323

*control* control condition, *pad* metatarsal pad orthosis, *cushioning* forefoot cushioning orthosis, *SD* standard deviation, *CI* confidence interval, *IQR* interquartile range

### Peak pressure in the total foot area

Considering the total foot area, peak pressures [kPa] were 364 ± 82 kPa (mean ± SD, lower-upper 95%-CI: 328–399) and median (IQR) 348 (310 to 440) for the control condition, 357 ± 70 (326–387) and 348 (303 to 393) for the metatarsal pad and 333 ± 81 (298–368) and 323 (270 to 363) for the cushioning orthosis.

### ICI sum score

Overall, participants reported medium to high comfort when wearing the different types of orthoses. Median values (IQR) of sum scores in comfort rating of the three different conditions are 50 (39 to 74) for the control condition, 49 (35 to 62) for the metatarsal pad and 64 (43 to 76) for the cushioning orthosis.

## Discussion

### Peak pressure

The purpose of this study was to compare the effect of forefoot cushioning and a metatarsal pad orthosis on the peak pressure in the forefoot of running healthy recreational runners. The principal finding was that the forefoot cushioning orthosis showed a lower peak pressure in the forefoot in comparison to the metatarsal pad orthosis and the control condition. These findings support previous research where it was shown that shock absorbing and cushioning insoles reduce peak pressure in the forefoot during walking and running in military personnel [34, 35]. Additionally, in the current literature about footwear and orthoses in sports medicine [48], forefoot cushioning orthoses are recommended for various conditions due to the positive experiences of clinicians but without any support of evidence including plantar pressure measurements.

The mean values of peak pressures for the forefoot area of this study are consistent with the current literature reporting peak pressures in runners without foot orthoses. One study examined the effect of foot type on in-shoe plantar pressure; the resulting pressure [kPa] in the forefoot was 304.6 ± 118.6 (mean ± SD) [9]. Another study analysed the effect of plantar fasciitis on pain and plantar pressure; the peak pressure [kPa] in the forefoot of the controls was 374.4 ± 96.4 [49]. A further study compared the plantar loads during treadmill and overground running; peak pressure [kPa] in the forefoot in treadmill running was 350.8 ± 82.3 [50]. These studies underline the validity of the current forefoot pressure data. Also, the total foot pressure data [kPa] of the mentioned studies above (305 to 381) were comparable to the current study (328 to 399), which further underlines the validity of measured data. Nonetheless, the methodologies of the mentioned literature differ from the present study mainly with respect to the running surface, running speed and running shoe or foot orthosis type. These differences may account for the small variations in total foot and forefoot pressure data in the findings of the studies mentioned above [17].

In contrast to earlier studies, however, the metatarsal pad used in the present study showed no significant pressure reduction in the forefoot in comparison to the two other conditions. These results differ from those of the current literature demonstrating that in running and walking individuals a metatarsal pad positioned just proximal to the metatarsal heads achieves optimal pressure reduction in the forefoot [20, 26, 29, 30, 51]. A possible explanation for this discrepancy might be the huge variability among the methodologies used (e.g., characteristics of the participants, footwear, and position of the metatarsal pad) and that the present study was the only one which used a full contact orthosis with an integrated pad (compared to isolated pads without full contact orthoses). In addition, the individual anatomical variations of the position of the metatarsal heads could influence the optimal positioning of metatarsal pads [31]. This point could not be considered in this study because prefabricated orthoses were used. By using customised orthoses, the metatarsal pads can be positioned individually according to the metatarsal head position. This presumably may lead to different results in pressure reduction.

### Comfort

In accordance with the presented results, it has been reported that recreational runners often wear foot orthoses for the treatment of running-related injuries, as a support or comfort device, and potentially to improve performance [52]. Authors pointed out that comfort is an important characteristic of footwear and may be influenced by impact perception and cushioning [53]. Hence, the participants in this study had to rate the perceived comfort of the three different conditions to obtain an impression of the individual subjective wear comfort of the orthoses. In this study, participants to a small extent (plus 15 points on ICI) preferred the forefoot cushioning orthosis over the two other conditions. These results are in accordance with the findings of an earlier study, in which healthy participants prioritised contouring or soft orthoses over hard orthoses [39, 54]. Each of the three tested conditions in this study was rated as comfortable. It can therefore be assumed that orthosis comfort did not influence the running sequences or the pressure measurements negatively.

### Limitations

Questions have been raised about the comparability of studies which have been conducted either with prefabricated or customised orthoses [16, 48]. However, a growing body of literature suggests that there seem to be only minor differences in the effects on plantar pressures between customised and prefabricated orthoses [25, 55–57]. By using prefabricated orthoses, the metatarsal pads could not be placed individually according to the metatarsal head location. This may lead to different results in forefoot pressure compared to customised orthoses with an individually located pad. The rationale of using prefabricated orthoses with identical surface designs (as presented in this study) was to guarantee a standardised measurement setting.

Several authors discussed the transferability of laboratory measurements made on a treadmill to overground running [50, 58–61]. In one study, it was concluded that treadmill running resulted in lower maximum pressures in the medial forefoot and toe regions [50]. Nevertheless, the treadmill setting allows optimal conditions for standardised measurements [58, 59]. In order to guarantee the reproducibility of the in-shoe plantar pressure measurement system pedar-x®-System, it is essential to control running speed [43]. Furthermore, authors pointed out that a familiarisation time of at least 6 min is required prior to data capturing; this recommendation has been respected in the present study [61]. Another limitation could be that the in-shoe plantar pressure measurement system provides a reliable measure of the vertical loading but no information about shear forces [32, 42, 62]. Hence, the effect of the 3D-shape of the tested orthoses could not be analysed precisely. However, in this study, the region of interest was the forefoot, which is a nearly flat area of the foot and therefore the “true” vertical component that is measured is assumed to be comparable to the normal force and can be assessed with sufficient accuracy. Moreover, the hypothesis of this study referred to the testing of relative differences in pressure values, which are likely to be more robust than absolute values [45]. Furthermore, in this study, only the peak pressure was reported as a pressure parameter because of its high correlation with the mean pressure and pressure–time integral [63–65]. The pedar-x®-System is used in foot orthoses and plantar pressure studies because it can be considered as the gold standard in plantar pressure assessment [24, 25].

The felt layer, which was fixed onto the surface of the orthoses used in this study for blinding reasons, could have reduced the cushioning effect of the forefoot cushioning orthosis. This aspect could not be considered in the results of this study but it can be assumed that it affected the results. Maybe a further reduction of peak pressure could be achieved without the layer that was integrated to blind the forefoot for all participants in this study.

Since the study was limited to runners with a heel-to-toe running pattern, no conclusion can be made for runners with a forefoot strike pattern [9]. In addition, all the runners participating in this study had normal-arched feet and therefore no statement can be made for runners with other foot postures.

Validity of the insole comfort index is limited because two criteria have not been met [39]. Firstly, subject-specific repeatability has not been established and secondly, the test has not been repeated over separate sessions [39]. The comfort rating of the orthoses was only used to exclude apparent discomfort during measurement. It was not a main outcome.

Despite these limitations, this study has considerable strengths to be mentioned. All the participants ran with the same neutral running shoe on a treadmill with a standardised running speed and therefore any speed or shoe-specific effect was avoided [66]. Considering the influence of foot type on in-shoe plantar pressure [9], the foot type of all participants was assessed and classified with the NNHt. As mentioned before in the methods section, all the participants in this study had normal-arched feet. Thus, the influence of differences in foot morphology should not have biased the results of this study. Furthermore, to control for possible sex-related differences in gait biomechanics, a heterogeneous group of female and male runners was recruited for this study [67].

## Conclusions

It is concluded that in contrast to the foot orthoses with a metatarsal pad, the forefoot cushioning orthosis was able to achieve a significant reduction of peak pressure in the forefoot of recreational runners. Consequently, prefabricated orthoses with forefoot cushioning reduce peak pressure in the forefoot more than prefabricated orthoses with an incorporated metatarsal pad. Therefore, cushioning should be considered if pressure reduction is the primary aim in runners with normal height arches.

Further research should investigate if patients with a forefoot-related overuse injury (e.g., metatarsalgia) benefit from intervention using foot orthoses with forefoot cushioning in prospective studies.

## Additional file

Additional file 1: The dataset with the raw data of peak pressures for the total foot and the forefoot. (XLSX 39 kb)

## Abbreviations

BMI: Body mass index
C: Control orthosis
CI: Confidence interval
FC: Forefoot cushioning orthosis
ICI: Insole comfort index
IQR: Interquartile-range
MH: Metatarsal head
MP: Metatarsal pad orthosis
n: Number (of participants)
NNHt: Normalised navicular height truncated
p: p-value to express significance
SD: Standard deviation.
